# Toll-like receptor 2–dependent endosomal signaling by *Staphylococcus aureus* in monocytes induces type I interferon and promotes intracellular survival

**DOI:** 10.1074/jbc.RA119.009302

**Published:** 2019-09-26

**Authors:** Jana Musilova, Michelle E. Mulcahy, Marieke M. Kuijk, Rachel M. McLoughlin, Andrew G. Bowie

**Affiliations:** School of Biochemistry and Immunology, Trinity Biomedical Sciences Institute, Trinity College Dublin, Dublin 2, Ireland

**Keywords:** innate immunity, host-pathogen interaction, Toll-like receptor (TLR), Staphylococcus aureus (S. aureus), monocyte, interferon, infectious disease, immune evasion, nonprofessional phagocyte

## Abstract

Pathogen activation of innate immune pattern recognition receptors (PRRs) such as Toll-like receptors (TLRs) stimulates cellular signaling pathways. This often leads to outcomes that contribute to pathogen clearance. Alternatively, activation of specific PRR pathways can aid pathogen survival. The human pathogen *Staphylococcus aureus* is a case in point, employing strategies to escape innate immune recognition and killing by the host. As for other bacteria, PRR-stimulated type I interferon (IFN-I) induction has been proposed as one such immune escape pathway that may favor *S. aureus*. Cell wall components of *S. aureus* elicit TLR2-dependent cellular responses, but the exact signaling pathways activated by *S. aureus*–TLR2 engagement and the consequences of their activation for the host and bacterium are not fully known. We previously showed that TLR2 activates both a cytoplasmic and an endosome-dependent signaling pathway, the latter leading to IFN-I production. Here, we demonstrate that *S. aureus* infection of human monocytes activates a TLR2-dependent endosomal signaling pathway, leading to IFN-I induction. We mapped the signaling components of this pathway and identified roles in IFN-I stimulation for the Toll-interleukin-1 receptor (TIR) adaptor Myd88 adaptor-like (Mal), TNF receptor-associated factor 6 (TRAF6), and IκB kinase (IKK)-related kinases, but not for TRIF-related adaptor molecule (TRAM) and TRAF3. Importantly, monocyte TLR2-dependent endosomal signaling enabled immune escape for *S. aureus*, because this pathway, but not IFN-I *per se*, contributed to intracellular bacterial survival. These results reveal a TLR2-dependent mechanism in human monocytes whereby *S. aureus* manipulates innate immune signaling for its survival in cells.

## Introduction

*Staphylococcus aureus* is an important human pathogen causing superficial skin infections as well as more severe invasive disease. The skin is a primary means of entry for deeper infections and bacterial invasion of the vascular system, leading to life-threatening conditions, such as endocarditis and bacteremia, which despite effective antimicrobial therapy are associated with significant mortality rates (1). Innate immunity comprises the first line of defense against invading microbes. Consequently, *S. aureus* has evolved an impressive arsenal of immune evasion strategies, which allows it to escape from innate immune recognition and killing by the host (2, 3). Deciphering how *S. aureus* interacts with and manipulates the host innate immune response will help toward developing novel host directed therapies much needed to treat invasive *S. aureus* infection, in particular in the face of rising antibiotic resistance (4). *S. aureus* invades nonprofessional phagocytes (5), thus facilitating its persistence in tissues. In addition, *S. aureus* can also survive within professional phagocytes, including neutrophils, monocytes, macrophages, and dendritic cells (6–9). Importantly, such cells may significantly contribute to the dissemination of *S. aureus* from the primary focus of infection to systemic loci (10). However, there is still only limited knowledge of which innate immune signaling pathways are engaged by *S. aureus* to facilitate this intracellular survival.

Anti-pathogen innate immune responses are triggered upon recognition of conserved pathogen-associated molecular patterns (PAMPs)^4^ by PRRs, including TLRs. TLR2 has been shown to play a key role in sensing cell wall structures of Gram-positive bacteria, including *S. aureus* lipoteichoic acid (LTA) and peptidoglycan (11). *S. aureus* mutants lacking lipoprotein expression do not activate TLR2 and fail to elicit proinflammatory responses in monocytes (12). The activation of TLRs initiates signaling cascades via adaptor proteins, which determine the type and duration of the host immune responses. Upon engagement of surface TLRs by ligands, the adaptor protein MyD88 oligomerizes and thus recruits interleukin-1 (IL-1) receptor-associated kinases (IRAKs), leading to formation of an oligomeric signaling complex termed the myddosome (13). Myddosome formation mediates phosphorylation of IRAKs and subsequent recruitment of tumor necrosis factor (TNF) receptor-associated factor 6 (TRAF6), which results in transforming growth factor β-activated kinase 1 (TAK1)-mediated activation of inhibitor of IκB kinase (IKK) complex consisting of IKKα, IKKβ, and IKKγ subunits (14, 15). IKK-dependent NF-κB activation then leads to transcription of proinflammatory cytokines such as TNFα. Importantly, clinical data from patients with MyD88 and IRAK4 deficiencies revealed a protective role for MyD88 and IRAK4 signaling during infections by pyogenic bacteria, including *S. aureus*, and cells from MyD88-deficient patients failed to respond to TLR2 ligands (16, 17). As well as MyD88, TLR2 requires MyD88 adaptor-like protein (Mal) (also called TIRAP) for TNFα induction in mouse bone marrow–derived macrophages (BMDMs) (18–20). Recently, Mal (TIRAP) deficiency in humans has also been linked to susceptibility to *S. aureus* infections (21).

TLRs, including TLR2, can signal from endosomes to drive interferon (IFN) regulatory factor (IRF) activation after ligand engagement, to elicit induction of type I IFNs (IFN-Is) and other IRF-dependent gene products (22). For example, we and others showed TLR2-mediated IFNβ production via MyD88-dependent IRF activation in response to pure TLR2 ligands in mouse BMDMs and that this induction of IFN-I depended on endolysosomal compartments (23, 24). In addition, we showed that TLR2-mediated IFN-I was Mal- and TRIF-related adaptor molecule (TRAM)-dependent in BMDMs (24) and that *S. aureus*-elicited IFNβ production required TRAM in BMDMs (24). Although IFN-Is have a well-defined antiviral role, their role during bacterial infections remains less understood. It is well-recognized that many bacteria including *S. aureus* elicit type I IFNs *in vitro* and *in vivo*, but the consequences of such IFN-I induction for the host *versus* the pathogen are often unclear and context-dependent (25, 26). As well as TRAM, there is also evidence that TLR2 can employ Toll/IL-1R domain–containing adaptor inducing IFNβ (TRIF) for endosome-dependent downstream signaling (27). Thus, in BMDMs, all four TIR adaptor proteins (MyD88, Mal, TRAM, and TRIF) have been implicated in TLR2 responses.

Monocytes represent one of the first lines of innate immune defense encountered by *S. aureus*, in particular during bloodstream infection, yet the strategies employed by *S. aureus* to manipulate these cells to gain a survival advantage has to date not been well established. Compared with mouse BMDMs, much less is known about how *S. aureus* engages TLR2 signaling pathways in human monocytes, in terms of which signaling components are involved and whether the TLR2 endosomal pathway leading to IFN-I induction is employed. Furthermore, the consequences of human monocyte TLR2 signaling and associated IFN-I induction for *S. aureus* intracellular survival have not been established. Here, we addressed these issues by examining TLR2-dependent responses to *S. aureus* using both the THP-1 human monocytic cell line and primary human monocytes from blood. We found that in both cell types, *S. aureus* infection led to TLR2-dependent induction of TNFα and type I IFN. In THP-1s, IFN-I but not TNFα induction depended on an endosomal signaling pathway that involved MyD88 and Mal, but surprisingly not TRAM. We also clarified the role of signaling components downstream of TIR adaptors in TNFα and IFN-I induction. Importantly, we show that the TLR2-dependent endosomal signaling pathway, but not IFN-I induction *per se*, enhances *S. aureus* intracellular survival, suggesting that engagement of TLR2-dependent endosomal signaling by *S. aureus* in human monocytes represents an immune escape pathway for the bacterium.

## Results

### S. aureus stimulates IFN-I production in human monocytes

**Figure 1.**
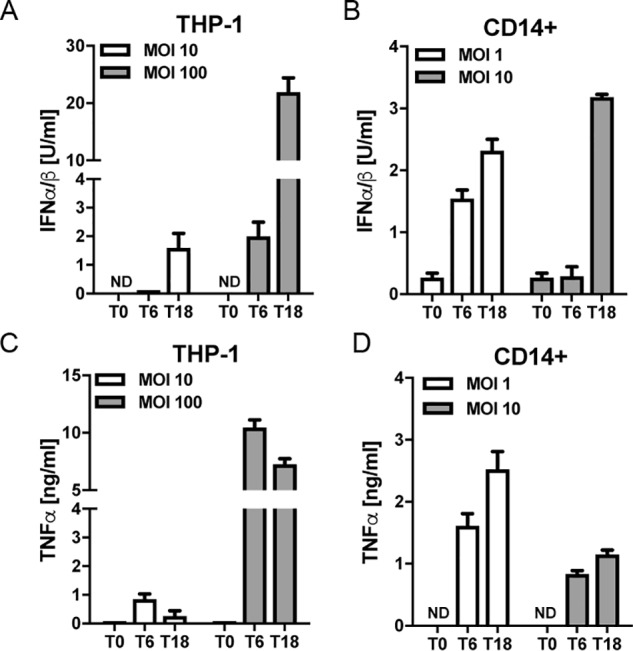
***S. aureus* induces type I IFN and TNFα production in human monocytes.** THP-1 monocytes (*A* and *C*) or primary human monocytes (*B* and *D*) were infected with *S. aureus* at the MOI indicated for 0, 6, and 18 h (*T0*, *T6*, and *T18*). Type I IFN bioactivity (*A* and *B*) or TNFα protein (*C* and *D*) in the supernatants was measured. Data are expressed as means ± S.D. (*error bars*) of triplicate samples and are representative of three independent experiments. *ND*, not detected.

We have previously shown in mouse BMDMs that TLR2-induced IFN-I utilized an endosomal signaling pathway that required the adaptors TRAM and Mal, whereas TLR2-induced TNFα was via a nonendosomal Mal-dependent pathway (24). To assess whether similar signaling pathways operated in human monocytes encountering live *S. aureus*, we first determined TNFα and IFN-I production from both the human monocytic cell line THP-1 and from blood-derived CD14^+^ monocytes in response to infection with *S. aureus. S. aureus* induced TNFα and IFN-I production in both THP-1 monocyte and primary human monocytes in a time-dependent manner (Fig. 1). A 6-h infection at a multiplicity of infection (MOI) of 100 in THP-1s gave a level of IFN-I production comparable with that of an 18-h infection at an MOI of 1 in primary monocytes (Fig. 1, *A* and *B*), and these infection regimes led to maximal TNFα production (Fig. 1, *C* and *D*). Thus, these conditions were used for future experiments.

### S. aureus–induced type I IFN is TLR2-dependent in human monocytes

**Figure 2.**
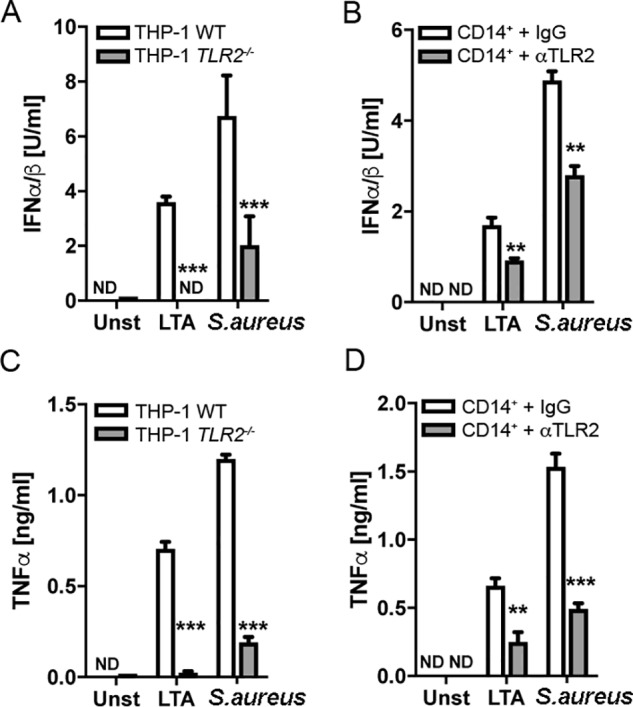
**TLR2 is required for LTA- and *S. aureus*-induced type I IFN and TNFα production in human monocytes.** WT or *TLR2*^−/−^ THP-1 monocytes were challenged with LTA (2.5 μg/ml) or *S. aureus* (MOI of 100) for 6 h (*A* and *C*). Alternatively, primary human monocytes were pretreated with αTLR2 antibody (2.5 μg/ml) or IgG1 isotype antibody (2.5 μg/ml) 1 h prior to challenge with LTA (2.5 μg/ml) or *S. aureus* (MOI of 1) for 18 h (*B* and *D*). Type I IFN bioactivity (*A* and *B*) and TNFα protein (*C* and *D*) in the supernatants were measured. Data are expressed as means ± S.D. (*error bars*). of triplicate samples and are representative of three independent experiments. *ND*, not detected; **, *p* < 0.01; ***, *p* < 0.001 (compared with infected WT cells (*A* and *C*) or IgG1 isotype–treated cells (*B* and *D*)).

TLR2 is known to be a major PRR for responding to *S. aureus*, and staphylococcal LTA is a key PAMP that binds TLR2 (11, 28). However, different cell types utilize different PRRs for specific *S. aureus* responses, and this is especially true of type I IFN induction (24, 26, 29, 30). Therefore, we tested the ability of LTA to induce IFN-I production by human monocytes, and the potential role of TLR2 in LTA– and *S. aureus*–stimulated IFN-I. To do this, we used a THP-1 TLR2 knockout cell line generated by CRISPR/Cas9 (31) as well as an anti-TLR2 blocking Ab to inhibit TLR2 responses in primary monocytes. Both LTA– and *S. aureus*–induced IFN-I production were significantly impaired in TLR2 knockout THP-1 cells compared with WT cells (Fig. 2*A*) and in primary human monocytes treated with anti-TLR2 Ab compared with cells treated with control IgG (Fig. 2*B*). This was also the case for TNFα production in response to either LTA or *S. aureus* (Fig. 2, *C* and *D*). Thus, TLR2 mediates both IFN-I and TNFα production after recognition of LTA as well as live *S. aureus*.

### A TLR2-dependent endosomal pathway is required for S. aureus–stimulated IFN-I

**Figure 3.**
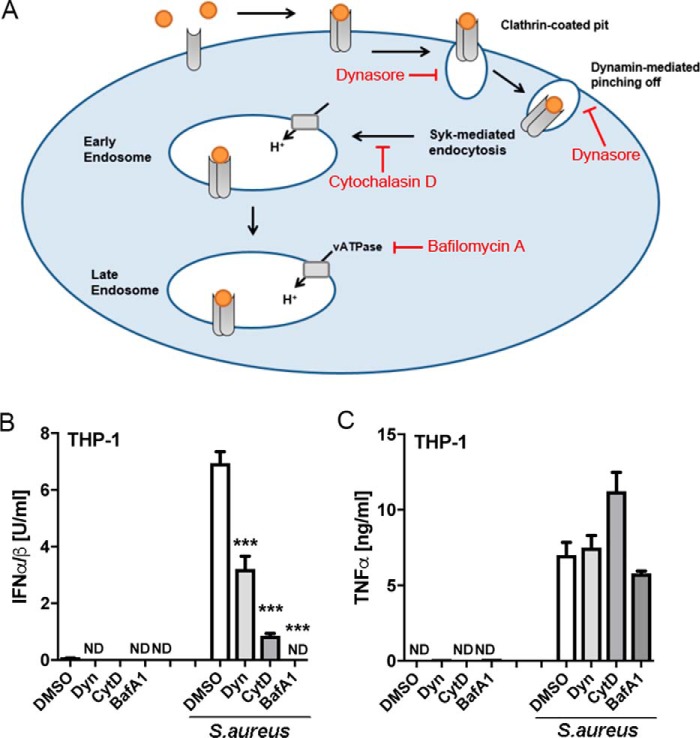
***S. aureus*-stimulated TLR2-dependent type I IFN but not TNFα requires an endosomal pathway in human monocytes.** *A*, *schematic* showing inhibitors of the endosomal pathway used, and their target. *B* and *C*, THP-1 monocytes were pretreated with 10 μm dynasore (*Dyn*), 10 μm cytochalasin D (*CytD*), 100 nm bafilomycin A1 (*BafA1*), or vehicle control (*DMSO*) for 1 h prior to infection with *S. aureus* (MOI of 100) for 6 h. Type I IFN bioactivity (*B*) and TNFα protein (*C*) in the supernatants were measured. Data are expressed as means ± S.D. (*error bars*) of triplicate samples and are representative of three independent experiments. *ND*, not detected; significant; ***, *p* < 0.001 (compared with infected DMSO-treated cells).

TLRs have been shown to signal from endosomes rather than from the plasma membrane to activate the IRFs required for IFN-I induction, and we previously showed that in mouse BMDMs, TLR2-stimulated IFN-I in response to bacterial lipoproteins required a bafilomycin A1–sensitive endosomal pathway (24). We therefore next tested whether *S. aureus*–induced IFN-I and TNFα in human monocytes required such an endosomal pathway. Thus, three inhibitors of the endosomal pathway were employed (Fig. 3*A*): dynasore, which inhibits formation of vesicles arising from clathrin-mediated endocytosis by targeting dynamin (32); cytochalasin D, which suppresses early endosome formation by inhibiting actin polymerization; and bafilomycin A1, which inhibits late endosome formation by blocking endosomal acidification by inhibiting the activity of the vacuolar H^+^ ATPase. Pretreatment of THP-1 cells with each of the endosomal inhibitors, prior to infection of cells with *S. aureus*, significantly inhibited IFN-I production compared with cells treated with vehicle (DMSO) (Fig. 3*B*). In stark contrast, TNFα production in response to *S. aureus* infection was not blocked by any of the inhibitors (Fig. 3*C*). As well as demonstrating that TNFα production was independent of endosomes, this result also showed that the inhibitors were not having a nonspecific inhibitory effect on cytokine production, for example due to toxicity. These data show that in human monocytes, detection of *S. aureus* by TLR2 leads to activation of at least two distinct signaling pathways, one involving endosomes (represented by IFN-I production) and one that is independent of endosomes (represented by TNFα production).

### Mal but not TRAM is required for S. aureus–induced IFN-I in human monocytes

**Figure 4.**
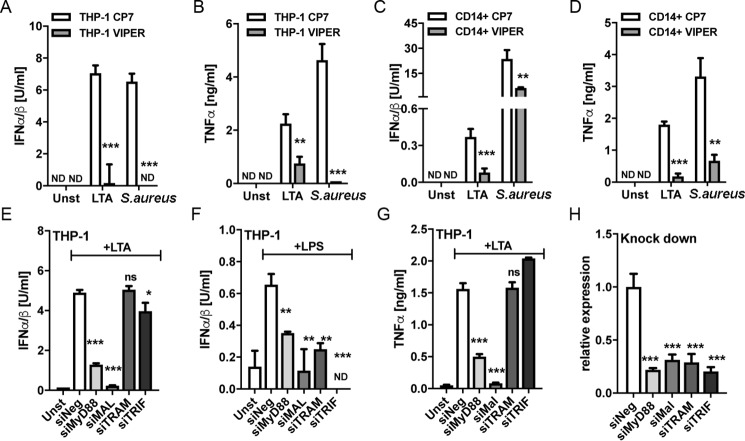
**Mal and not TRAM is required for LTA-stimulated type I IFN and TNFα production in human monocytes.** *A–D*, THP-1 cells (*A* and *B*) or primary human monocytes (*C* and *D*) were pretreated with 5 μm VIPER or control peptide (CP7) 1 h prior to stimulation with 2.5 μg/ml LTA (*A–D*) or infection with *S. aureus* at an MOI of 100 for 6 h (*A* and *B*) or an MOI of 1 for 18 h (*C* and *D*). *E–H*, THP-1 cells were electroporated with a 100 nm concentration of the indicated siRNAs for 48 h prior to stimulation with 2.5 μg/ml LTA (*E* and *G*) or 100 ng/ml LPS (*F*) for 6 h. The knockdown of siRNA target expression was confirmed by qRT-PCR using gene-specific primers (*H*). Type I IFN bioactivity and TNFα protein in the supernatants were measured as indicated. Data are expressed as means ± S.D. (*error bars*) of triplicate samples and are representative of three independent experiments. *ND*, not detected; *ns*, not significant; *, *p* < 0.05; **, *p* < 0.01; ***, *p* < 0.001 (compared with stimulated CP7-treated cells (*A–D*) or stimulated cells transfected with negative siRNA (*E–H*)).

Endosomal TLR signaling requires a so-called TIR sorting adaptor, namely Mal and/or TRAM, to activate IRFs and subsequently induce IFN-Is, whereas TLR2 and TLR4 signaling from the plasma membrane also require Mal (14, 24, 33). We therefore tested the potential role of specific TIR adaptor proteins in *S. aureus*–stimulated IFN-I production. VIPER is a peptide derived from the poxviral TIR antagonist protein A46, which inhibits Mal- and TRAM-dependent cellular responses in mouse BMDMs (34). Both LTA and *S. aureus* IFN-I and TNFα responses in THP-1s and primary monocytes were significantly inhibited by pretreatment of cells with VIPER, compared with control peptide (Fig. 4, *A–D*), consistent with the notion that Mal and/or TRAM are required for both the cytoplasmic TNFα response and the endosomal IFN-I response pathways. Next, we used siRNA targeting specific TIR adaptors to delineate exactly which TIR adaptors were required for each pathway in THP-1 cells. siRNAs targeting MyD88, Mal, TRAM, and TRIF were employed and were shown to be effective in reducing expression of their respective targets (Fig. 4*H*). LTA-stimulated IFN-I was significantly inhibited by MyD88 and Mal targeting, whereas TRAM siRNA had no effect, and TRIF siRNA had a small but significant effect, on IFN-I production. The lack of a role for TRAM was surprising, given that TLR2-stimulated IFN-I in mouse BMDMs was TRAM-dependent (24), so as a positive control, we showed that the TRAM siRNA was effective in reducing TLR4-stimulated IFN-I in the same cell type (Fig. 4*F*). Thus, TLR2 utilizes different sorting adaptors for IFN-I induction in human monocytes compared with mouse BMDMs. As expected, siRNA targeting MyD88 and Mal, and not TRAM and TRIF, significantly inhibited the LTA-stimulated cytoplasmic TNFα response (Fig. 4*G*). Because the combination of *S. aureus* infection and siRNA treatment of cells was toxic, it was not possible to employ siRNA to directly examine TIR adaptor involvement in live *S. aureus* responses. These results suggest that Mal, and not TRAM, is the key sorting adaptor for TLR2 endosomal responses in human monocytes and that both the TLR2 cytoplasmic and endosomal signaling pathways utilize Mal and MyD88 in these cells.

### The myddosome and TRAF6 are involved in the TLR2-dependent endosomal pathway

**Figure 5.**
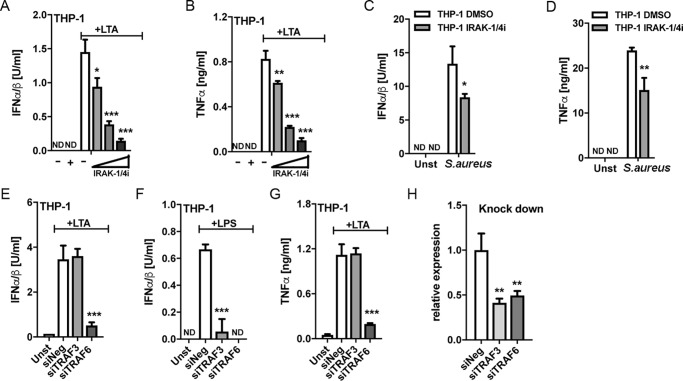
**IRAKs and TRAF6, but not TRAF3, are required for LTA-stimulated type I IFN and TNFα production in human monocytes.** *A–D*, THP-1 monocytes were pretreated with IRAK-1/4 inhibitor (+, 2 μm; *wedge*, 0.1, 1, and 2 μm; or 2 μm in *C* and *D*) or equivalent amount of DMSO (−) for 1 h prior to stimulation with 2.5 μg/ml LTA (*A* and *B*) or infection with *S. aureus* at an MOI of 100 (*C* and *D*) for 6 h. *E–H*, THP-1 monocytes were electroporated with the indicated siRNAs for 48 h prior to stimulation with 2.5 μg/ml LTA (*E* and *G*) or 100 ng/ml LPS (*F*) for 6 h. The knockdown of siRNA target expression was confirmed by qRT-PCR using gene-specific primers (*H*). Type I IFN bioactivity and TNFα protein in the supernatants were measured as indicated. Data are expressed as means ± S.D. (*error bars*) of triplicate samples and are representative of three independent experiments. *ND*, not detected; **, *p* < 0.01; ***, *p* < 0.001 (compared with stimulated DMSO-treated cells (*A–D*) or stimulated cells transfected with negative siRNA (*E–H*)).

We next defined which TLR signaling components downstream of Mal and MyD88 were involved in human monocyte TLR2-dependent responses. After stimulation by upstream TLRs, MyD88 forms the myddosome, a multiprotein complex that also contains IRAKs and mediates activation of downstream signaling (13). Consistent with a role for the myddosome in both the cytoplasmic and endosomal signaling pathways, treatment of cells with a small molecule that inhibits IRAK1 and IRAK4 suppressed LTA and *S. aureus*–stimulated TNFα and also IFN-I (Fig. 5, *A–D*).

The myddosome typically activates the TRAF6 ubiquitin ligase to induce proinflammatory cytokines (15). TRAF3 is required for endosomal TLR4 and TLR3 signaling via TRIF to IFN-I production (35), but whether TRAF3 is also involved in endosomal TLR2-induced IFN-Is is unknown. Thus, we used siRNA to examine whether TRAF6 and/or TRAF3 were required for LTA-stimulated IFN-I in human monocytes. Interestingly, TRAF6 siRNA, but not TRAF3 siRNA, strongly inhibited LTA-stimulated IFN-I (Fig. 5*E*). In parallel, TRAF3 siRNA was effective in inhibiting LPS-stimulated IFN-I, as would be expected (Fig. 5*F*), whereas LTA-stimulated TNFα was TRAF6- but not TRAF3-dependent (Fig. 5*G*). Fig. 5*H* shows that TRAF3 and TRAF6 siRNA were effective in silencing their targets.

This result suggests that, unlike the case for TLR4 signaling, TRAF3 does not mediate induction of IFN-Is downstream of TLR2 in human monocytes. Instead, TRAF6 activated by the myddosome mediates TLR2-dependent type I IFN induction.

### S. aureus–induced type I IFN production involves both IKKβ and IKK-related kinases

**Figure 6.**
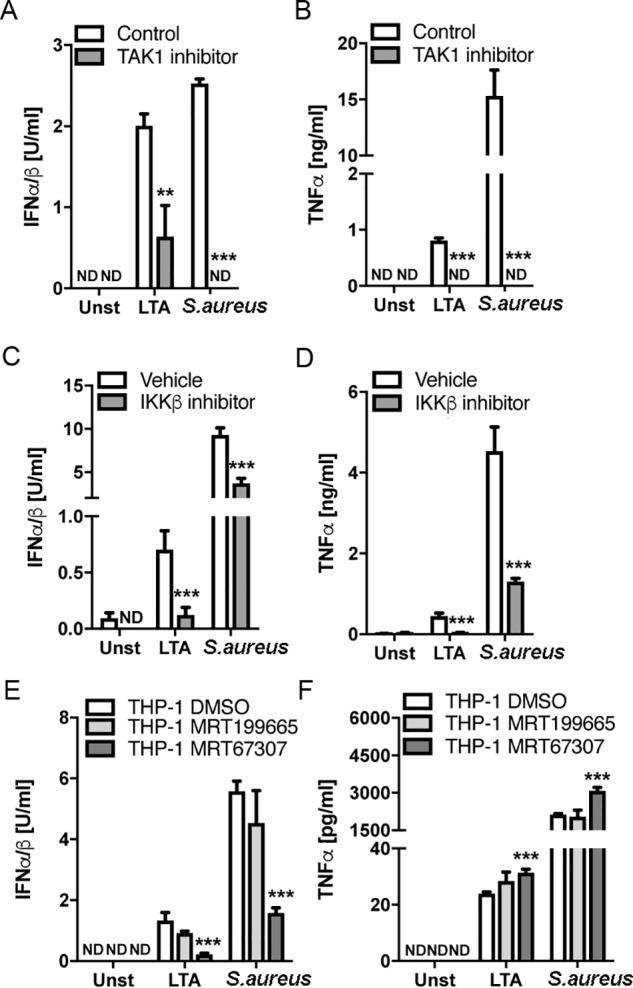
**Role of TAK1 and IKK family members in LTA– and *S. aureus*–stimulated type I IFN production in human monocytes.** *A* and *B*, THP-1 monocytes were pretreated with 1 μm TAK1 inhibitor ((5*Z*)-7-oxozeaenol) or control compound ((5*Z*)-7-zeaenol) for 1 h prior to stimulation with 2.5 μg/ml LTA or infection with *S. aureus* at an MOI of 100, for 6 h. *C* and *D*, THP-1 monocytes were pretreated with 10 μm IKKβ inhibitor (BI605906) or an equivalent amount of vehicle (DMSO) for 1 h prior to stimulation with 2.5 μg/ml LTA or infection with *S. aureus* at an MOI of 100 for 6 h. *E* and *F*, THP-1 monocytes were pretreated with the TBK1/IKKϵ inhibitor MRT67307 (2 μm), control compound MRT199665 (1 μm), or equivalent amount of vehicle (DMSO) for 1 h prior to stimulation with 2.5 μg/ml LTA or infection with *S. aureus* at an MOI of 100 for 6 h. Type I IFN bioactivity and TNFα protein in the supernatants were measured as indicated. Data are expressed as means ± S.D. (*error bars*) of triplicate samples and are representative of three independent experiments. *ND*, not detected; **, *p* < 0.01; ***, *p* < 0.001 (compared with stimulated cells treated with control compound (*A* and *B*) or DMSO vehicle (*C–F*)).

TAK1 is a key target molecule downstream of MyD88 and TRAF6 in TLR signaling, and consistent with the role of MyD88 and TRAF6 in the TLR2 endosomal pathway in THP-1, LTA– and *S. aureus*–stimulated IFN-I was strongly diminished after inhibition of TAK1 by (5*Z*)-7-oxozeaenol compared with the control compound (5*Z*)-7-zeaenol (Fig. 6*A*). As expected, TAK1 inhibition also blocked TNFα production via the cytoplasmic pathway (Fig. 6*B*).

We next assessed the role of the IKKs in human monocyte TLR2 signaling by *S. aureus*. During TLR signaling, TAK1 forms a complex that facilitates recruitment of the canonical IKK complex containing IKKα and IKKβ (36). TAK1 then phosphorylates IKKβ, leading to NF-κB activation and transcription of proinflammatory cytokines. MyD88-independent signaling, for example via TRIF, activates the IKK-related kinases IKKϵ and TRAF family member–associated NF-κB activator–binding kinase 1 (TBK1), which phosphorylate IRFs, leading to transcription of IFN-Is (37). Cross-talk also occurs between TBK1 and IKKβ in some pathways (38). To clarify which IKKs are involved in the endosomal TLR2 pathway to IFN-I induction, we utilized a specific inhibitor of IKKβ, namely BI605906 (38). We also used a pair of compounds to examine the role of IKK-related kinases, MRT67307 and MRT199665. Inhibition of a response by MRT67307, but not MRT199665 indicates a role for IKKϵ or TBK1 in that response (38). This showed that both IKKβ and IKK-related kinases were required for IFN-I production, because both the IKKβ inhibitor and the IKK-related kinase inhibitor reduced LTA– or *S. aureus*–stimulated IFN-I by more than 50% (Fig. 6, *C* and *E*). For the cytoplasmic pathway measured by TNFα production, only the IKKβ and not the IKK-related kinase inhibitor suppressed the LTA and *S. aureus* response (Fig. 6, *D* and *F*). MRT67307 treatment led to a subtle but significant increase in TNFα production (Fig. 6*F*), likely because in some contexts, IKK-related kinases are able to inhibit canonical IKKs and thus reduce transcription of NF-κB–dependent genes (39). Collectively, these results suggest that both IKKβ and IKK-related kinases are employed by TLR2 signaling in response to *S. aureus* in human monocytes.

### TLR2 contributes to S. aureus intracellular survival in human monocytes

**Figure 7.**
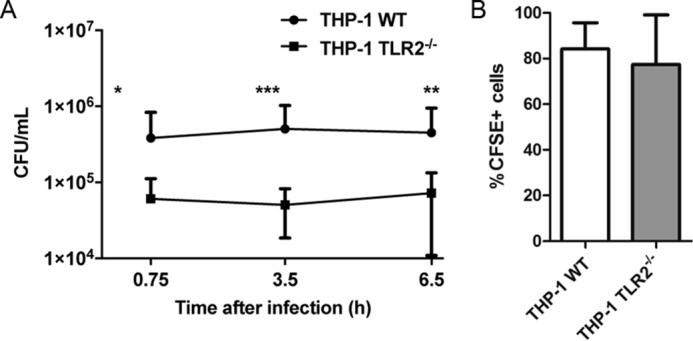
**TLR2 contributes to *S. aureus* intracellular survival in human monocytes.** *A*, WT or *TLR2*^−/−^ THP-1 monocytes were infected with *S. aureus* at an MOI of 100 for 30 min. Monocytes were then treated with gentamicin and lysed at the indicated times post-infection, and intracellular bacteria were enumerated. Data are expressed as mean cfu/ml ± S.D. (*error bars*), pooled from six independent experiments; *, *p* < 0.05; **, *p* < 0.01; ***, *p* < 0.001 as determined by two-way ANOVA of log-transformed data with Bonferroni post-tests. *B*, WT or *TLR2*^−/−^ THP-1 monocytes were infected with CFSE-labeled *S. aureus* strain SH1000 at an MOI of 100 for 30 min and then treated with gentamicin for 15 min. Cells were fixed and analyzed by flow cytometry. Data are expressed as mean percentage of CFSE-positive cells ± S.D., pooled from three individual experiments.

Having showed that TLR2 engagement by *S. aureus* in human monocytes leads to TNFα and IFN-I production and defined the signaling components required, we next assessed the contribution of TLR2 to intracellular survival of *S. aureus* in human monocytes. This was of interest because, so far, research conducted on the survival or killing of *S. aureus* within myeloid cells has focused primarily on neutrophils (6, 40) and macrophages (8, 41). WT and TLR2 knockout THP-1 cells were infected with *S. aureus* (MOI = 100) for 30 min, at which time cells were treated with gentamicin to kill nonphagocytosed extracellular bacteria. Intracellular bacteria were then quantified 15 min (45 min post-infection), 3 h (3.5 h post-infection), or 6 h (6.5 h post-infection) after gentamicin addition. Intracellular survival of *S. aureus* within TLR2 knockout cells was significantly reduced as compared with WT cells at each time point (Fig. 7*A*). At 45 min post-infection with *S. aureus*, there was already a significant reduction in the number of intracellular bacteria within TLR2 knockout THP-1 cells compared with WT cells. Importantly, however, when we compared phagocytic uptake of bacteria by the WT and TLR2 knockout cells after 45 min of infection, no differences were observed (Fig. 7*B*), confirming that the absence of TLR2 signaling does not impair phagocytosis of *S. aureus* by the THP-1 cells; however, it appears that *S. aureus* engages TLR2 signaling in these cells to facilitate its intracellular survival.

### The TLR2 endosomal pathway, but not IFN-I, contributes to intracellular survival

**Figure 8.**
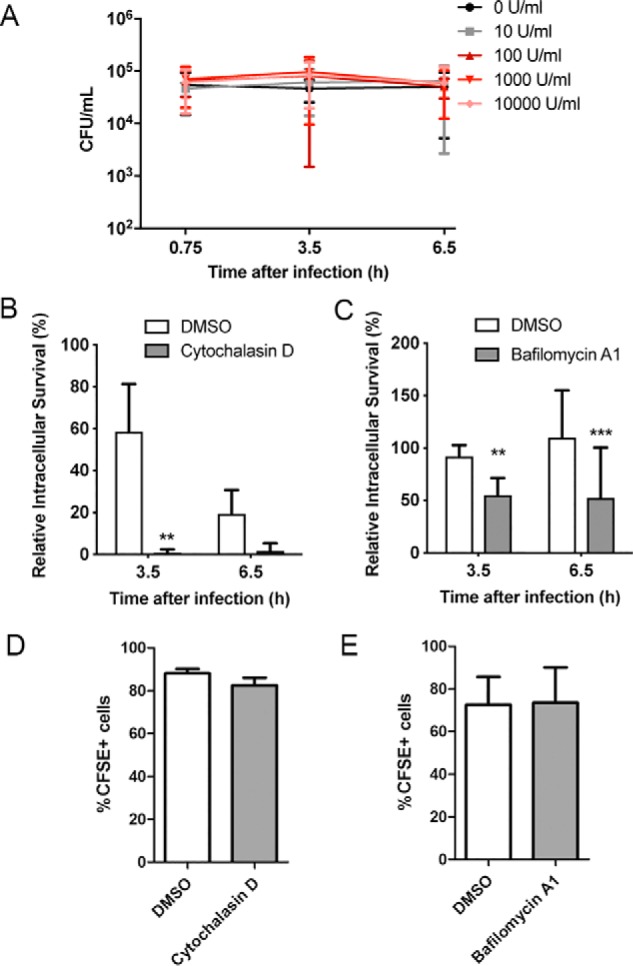
**The TLR2-endosomal pathway, but not type I IFN, contributes to *S. aureus* intracellular survival in human monocytes.** *A–C*, THP-1 monocytes were pretreated with 10–10,000 units/ml IFNα for 16 h (*A*), 10 μm cytochalasin D for 1 h (*B*), or 100 nm bafilomycin A1 for 1 h (*C*) prior to infection with *S. aureus* at an MOI of 100 for 30 min. Monocytes were then treated with gentamicin and lysed at the indicated times post-infection, and intracellular bacteria were enumerated. Data are expressed as mean cfu/ml ± S.D. (*error bars*) (*A*) or mean percentage of intracellular survival ± S.D. compared with 0 h (*B* and *C*), pooled from 3–4 independent experiments; **, *p* < 0.01; ***, *p* < 0.001 as determined by two-way ANOVA with Bonferroni post-tests. *D* and *E*, THP-1 monocytes were pretreated with 10 μm cytochalasin D (*D*) or 100 nm bafilomycin A1 (*E*) for 1 h prior to infection with CFSE-labeled *S. aureus* at an MOI of 100 for 30 min and then treated with gentamicin for 15 min. Cells were fixed and analyzed by flow cytometry. Data are expressed as mean percentage of CFSE-positive cells ± S.D., pooled from three individual experiments.

Because TLR2 contributed to intracellular survival of *S. aureus* within THP-1 cells, we wondered whether this involved the cytoplasmic or endosomal pathways engaged by *S. aureus* recognition by TLR2 and also whether IFN-I may be directly involved in promoting intracellular survival of *S. aureus*. To address this, cells were treated with a log dose range of IFNα, from 10 to 10,000 units/ml, for 16 h prior to infection with *S. aureus* followed by gentamicin treatment to kill any nonphagocytosed extracellular bacteria. IFNα treatment had no significant effect on the levels of *S. aureus* surviving intracellularly within THP-1 cells at any of the time points or IFN concentrations tested (Fig. 8*A*). Despite the lack of effect of IFN-I on intracellular survival, we wondered whether other cell-intrinsic outcomes regulated by the TLR2-dependent endosomal pathway might contribute to the role of TLR2 in intracellular survival. To test this, we again utilized cytochalasin D and bafilomycin A, two compounds that inhibit endosomal signaling after TLR2 activation (Fig. 3, *A* and *B*) but had no effect on the cytoplasmic pathway (Fig. 3*C*). THP-1 cells were treated with these inhibitors for 1 h and then infected with *S. aureus* (MOI = 100) for 30 min before gentamicin treatment to remove any nonphagocytosed extracellular bacteria. Intracellular survival was then assessed at 3.5 and 6.5 h post-infection. Inhibition of endosomal TLR2 signaling using either inhibitor significantly reduced intracellular survival of *S. aureus* within THP-1 cells (Fig. 8, *B* and *C*). Importantly, the rate of phagocytosis of *S. aureus* by the THP-1 cells was not affected by inhibitor treatment (Fig. 8, *D* and *E*). Taken together, these data suggest that *S. aureus* manipulates the endosomal TLR2 signaling pathway to promote its survival in human monocytes.

## Discussion

TLRs have a critical role in alerting immune cells to the presence of pathogens, whereas pathogens can also manipulate intracellular TLR responses for their benefit. Much has been learned from studies using TLR knockout mice and mouse BMDMs about the intracellular signaling events that occur when TLRs encounter pathogens. Further, it has been known for some time that TLR2 has a major role in detection of Gram-positive bacteria, such as *S. aureus*, and that LTA is a major *S. aureus* PAMP that is recognized by TLR2 (11, 42). However, much less is known about the relationship between TLR2 and *S. aureus* in human monocytes, although this cell type likely contributes significantly to the host response to *S. aureus* during bloodstream infection, where these cells are first line responders to infection. It was originally thought that TLR2 signaling proceeded only via a cytoplasmic pathway involving MyD88 and Mal, leading to induction of mainly NF-κB–dependent genes, such as TNFα (18, 19). This was in contrast to TLR4, which was shown to employ both a cytoplasmic MyD88/Mal-dependent pathway similar to TLR2, but also a TRAM/TRIF-dependent endosomal pathway leading to IRF3 activation and induction of IFNβ (22). More recently, we and others showed that TLR2 actually also employs an endosomal pathway to activate IRFs and induce IFN-Is (23, 24) and that in mouse BMDMs stimulated with bacterial lipoprotein TLR2 ligands, IRF activation required both Mal and TRAM (24). However, whether a similar TLR2 pathway operated for *S. aureus* infection of human monocytes, and what signaling molecules downstream of the TIR adaptors were involved in the TLR2 endosomal pathway, were not known; nor were the consequences of TLR2 pathway engagement to *S. aureus* persistence inside cells. Here, we have addressed these issues by defining and mapping cytoplasmic and endosomal arms of the TLR2 pathway that are activated in response to *S. aureus* in human THP-1 and primary monocytes and examining their potential role in *S. aureus* intracellular survival in monocytes.

*S. aureus* infection of cells leads to production of both pro-inflammatory cytokines, such as TNFα, but also the induction of type I IFNs, such as IFNβ, and here we found that TLR2 had a role in both responses in human monocytes. Disruption of TLR2 by CRISPR/Cas9 in THP-1s or use of an anti-TLR2 blocking antibody in primary human monocytes significantly suppressed, but did not completely ablate, IFN-I production in response to *S. aureus* infection. Hence, TLR2 played a major, but not exclusive, role in IFN-I induction in these human monocytes, likely largely via TLR2 sensing of LTA, because LTA-stimulated IFN-I (and TNFα) were completely ablated in THP-1s lacking TLR2. Because the LTA response was not completely blocked by the anti-TLR2 antibody in primary monocytes, this suggests that the antibody may not have been 100% efficient in preventing TLR2 signaling in the primary cells. Hence, some of the remaining *S. aureus* cytokine production in the presence of the antibody may still have been mediated by TLR2. Other PRRs that may contribute to IFN-I induction by *S. aureus* in THP-1s and primary monocytes include TLR8 sensing of *S. aureus* mRNA (43), cGAS-STING sensing of bacterial DNA (30), and STING sensing of *S. aureus* c-di-AMP (29).

Using specific kinase inhibitors and siRNA, we mapped the TLR signaling proteins required for both the endosomal and cytoplasmic arms of the human monocytic TLR2 response to *S. aureus*, represented by IFN-I and TNFα production, respectively. For TNFα production, we confirmed the role of proteins shown to be involved in TLR2 responses in other cell types, namely Mal, MyD88, IRAKs, TRAF6, TAK1, and IKKβ.

However, for IFN-I production via endosomal signaling, it was unclear exactly which components would be required because much less is known about endosomal TLR2 signaling, especially in human cells. For the LTA stimulation via TLR2, we found that the TIR adaptor TRAM had no role in the IFN-I response, but rather, the alternative sorting adaptor Mal controlled this pathway in human monocytes. This strongly suggests that live *S. aureus* also signals via Mal and not TRAM to IFN-I induction in these cells. The lack of a role for TRAM was surprising, given that TLR2-stimulated IFN-I in mouse BMDMs was TRAM-dependent. Therefore, we confirmed that TLR4 signaling did indeed use TRAM in these cells. Thus, TLR2 utilizes different sorting adaptors for IFN-I induction in human monocytes compared with mouse BMDMs. Interestingly, Mal (TIRAP) deficiency has recently been linked to severe childhood staphylococcal disease (21), whereas the same study found no evidence of a similar link with TRAM or TRIF. Hence, to date, deficiencies in MyD88, IRAK4, and Mal (TIRAP), but not TRAM and TRIF, have been linked to increased human susceptibility to *S. aureus,* consistent with the repertoire of TLR-signaling proteins that we found are involved in human TLR2 responses to the bacterium. Previously, Nilsen *et al.* (27) showed a role for TRIF in TLR2-stimulated CCL5 (via endosomal signaling), but not TNFα, in mouse peritoneal macrophages, and although we did find a small but significant effect of TRIF siRNA on LTA-stimulated IFN-I (but not TNFα) in THP-1s here, the contribution of MyD88 and Mal was much more marked.

These results imply that like TLR9, and in contrast to TLR3 and TLR4, which use TRIF, TLR2 endosomal signaling in human monocytes mediates myddosome formation for downstream signaling. Also consistent with this difference from TLR3 and TLR4 is the fact that TRAF6 and not TRAF3 was required for LTA-dependent IFN-I, suggesting that live *S. aureus* also utilizes TRAF6 for IFN-I induction. Formal confirmation of the role of both Mal and TRAF6 in live *S. aureus*–stimulated IFN-I production will need to be demonstrated in human monocytes lacking Mal and TRAF6. Overall, these data together with previous studies suggest that Mal can mediate myddosome formation at the plasma membrane for TLR2 and TLR4 cytoplasmic signaling and at the endosome for TLR2 and TLR9 endosomal signaling. Interestingly, as well as a role for IKKβ, which is known to be activated downstream of TAK1 after myddosome formation, our data also show a role for IKK-related kinases in TLR2-stimulated IFN-I but not TNFα production, consistent with the fact that TBK1 has recently been shown to be activated by myddosome formation (15).

Although we defined a clear role of TLR2 in IFN-I induction in human monocytes, we failed to observe any cell-intrinsic role of IFN-I on *S. aureus* in monocytes, because direct treatment of infected cells with IFNα did not affect intracellular survival of the bacterium. This does not exclude a contribution of monocyte-elicited IFN-I on other cells in the context of a human *S. aureus* infection. Type I IFNs have been reported to protect human lung epithelial cells during *S. aureus* infection *in vitro* (44), to enhance clearance of *S. aureus* by mouse myeloid cells *in vitro* (45) and to reduce bacterial load during mouse cutaneous infection *in vivo* (45), thus suggesting a protective role of type I IFNs during *S. aureus* infection. In contrast, mice deficient in type I IFN receptor (*Ifnar*) were shown to have improved clearance of *S. aureus* from the lungs (46). Similarly, *Ifnar*^−/−^ mice exhibited reduced mortality during *S. aureus* pulmonary infection compared with their WT littermates (47), suggesting a pathogenic role of type I IFNs. Further, it has been suggested that strain-specific differences in the ability of *S. aureus* to induce IFN-I are responsible for the increased pathogenicity of some strains (26). Thus, the relationship between *S. aureus* infection and host IFN-I production is complex and may vary between bacterial strains and host species.

Importantly, however, we did find that TLR2 affects cell intrinsic responses to *S. aureus* in human monocytes, because deletion of TLR2 reduced intracellular survival while not affecting the initial phagocytosis of *S. aureus* by monocytes. Specifically, it was the TLR2-dependent endosomal signaling pathway that was involved here, because when this arm of TLR2 signaling was blocked with cytochalasin D or bafilomycin A1, intracellular survival (but not phagocytic uptake) was significantly reduced. These data suggest that *S. aureus* activates the TLR2 endosomal signaling pathway as an immune escape mechanism to suppress its killing in human monocytes. How exactly TLR2-dependent endosomal signaling affects intracellular survival remains to be elucidated, but our data suggest that IFN-I induction is not involved. Alternatively, *S. aureus* is known to activate host kinases such as p38 MAPK to boost intracellular survival (48), whereas bacteria-stimulated altered host cellular metabolism may also be involved (49), and both p38 MAPK activation and altered metabolism are known outcomes of TLR2 signaling via MyD88, the latter of which requires TBK1 (15).

In summary, here we show that *S. aureus* activates a TLR2-dependent endosomal signaling pathway in human monocytes that utilizes Mal, MyD88, TRAF6, and IKK-related kinases. The consequences of activation of this pathway are two seemingly independent effects, namely IFN-I production and enhanced intracellular survival. Thus, activation of this pathway may be a strategy employed by *S. aureus* to manipulate the host to promote intracellular survival while simultaneously driving type I IFN responses. These results contribute to deciphering the mechanisms by which *S. aureus* manipulates innate immune signaling pathways to gain a survival advantage that is important in identifying novel targets for new host-directed therapies urgently required to treat antibiotic-resistant *S. aureus* infections.

## Experimental procedures

### Cells

THP-1 cells were purchased from European Collection of Authenticated Cell Cultures (ECACC). TLR2 knockout THP-1 cells generated using CRISPR/Cas9 were a gift from Prof. Veit Hornung (Ludwig-Maximilians-Universität München, Munich, Germany) and were described previously (31). THP-1 cells were grown in RPMI 1640 medium GlutaMAX^TM^ containing 10% (v/v) FCS, 100 units/ml penicillin, and 100 μg/ml streptomycin. The THP-1 CRISPR knockout cells were grown in RPMI 1640 medium GlutaMAX^TM^ containing 10% (v/v) FCS, 10 μg/ml ciprofloxacin, and 1 mm sodium pyruvate.

Peripheral blood mononuclear cells (PBMCs) were obtained from the Irish Blood Transfusion Centre. Ethical approval was obtained from the TCD Faculty of Health Sciences Ethics Committee for experiments involving PBMCs. Blood for PBMC isolation was obtained in the form of buffy coat packs. PBMCs were isolated by density gradient using lymphoprep (Axis-Shield Density Gradient Media). CD14^+^ cells were positively selected using CD14^+^ microbeads and LS columns for MACS cell separation (Miltenyi Biotec) according to the manufacturer's instructions and cultured in RPMI 1640 medium GlutaMAX^TM^ containing 10% (v/v) FCS.

HEK-Blue^TM^ IFN-α/β cells (InvivoGen) were grown in Dulbecco's modified Eagle's medium GlutaMAX^TM^ containing 10% (v/v) FCS, 50 units/ml penicillin, 50 μg/ml streptomycin, 30 μg/ml blasticidin, 100 μg/ml Normocin^TM^, and 100 μg/ml Zeocin^TM^.

### Bacterial strains

*S. aureus* strain SH1000 was described previously (50). Bacteria were cultivated from frozen stocks for 24 h at 37 °C on tryptic soy agar plates. *S. aureus* inoculum was prepared in PBS, and the absorbance of the suspension was measured at 600 nm and was adjusted to *A*_600_ = 0.43–0.45, which was estimated to contain 4 × 10^8^ cfu/ml.

### Ligands, reagents, and inhibitors

Purified LTA-SA was from Invivogen. Human IFNα was from PBL Assay Science. Bafilomycin A1, cytochalasin D, and dynasore were purchased from Sigma-Aldrich. Neutralizing anti-human TLR2 antibody and IgG1 isotype control were purchased from eBioscience. VIPER and CP7 peptides were from Novus Biologicals. (5*Z*)-Zeaenol, (5*Z*)-7-oxozeaenol, and the IRAK-1/4 inhibitor were purchased from Merck Millipore. BI605906, MRT67307, and MRT199665 compounds were a gift from Prof. Philip Cohen (University of Dundee) and were described previously (38, 39).

### Stimulation and infection of cells

Cells (5 × 10^5^ cells/ml) were seeded into 96-well plates in complete medium prior to stimulation with LTA (2.5 μg/ml) or LPS (100 ng/ml). Alternatively, the cells (1 × 10^6^ cells/ml) were seeded into 24-well plates in medium without antibiotic prior to infection with *S. aureus*.

THP-1 monocytes were infected with live *S. aureus* at MOI = 100 unless otherwise indicated, and primary CD14^+^ monocytes were infected with live *S. aureus* at MOI of 1 or 10 for the specific times indicated in the figure legends. After 2 h, medium was replaced with medium containing gentamicin (200 μg/ml) to kill extracellular bacteria. The supernatants were then collected at the indicated times and assayed for cytokine production.

### Cytokine analysis

TNFα protein in supernatants was detected using the TNFα ELISA kit (R&D) according to the manufacturer's instructions. Bioactive type I IFNs in the supernatants were detected using the HEK-Blue IFNα/β bioassay (InvivoGen). Briefly, supernatants and IFNα standard were diluted in the test medium (Dulbecco's modified Eagle's medium, 10% fetal calf serum, 50 units/ml penicillin, 50 μg/ml streptomycin, 100 μg/ml normocin). HEK-Blue IFNα/β cells (2.8 × 10^5^ cells/ml) were seeded to 96-well plates containing supernatant, standard, and blank. Following 24-h incubation at 37 °C, secreted SEAP was detected by QUANTI-Blue, and absorbance was measured at 620 nm.

### siRNA silencing

RNA silencing was performed using the Neon® transfection system (Invitrogen). Targeting or nontargeting siRNA at the indicated concentration was added to the THP-1 cells (2 × 10^6^) resuspended in R Buffer. The suspension was collected in a 100-μl Neon® tip using a Neon® pipette. The pipette was placed into the Neon® electroporation chamber filled with E2 buffer. The cells were then electroporated using preset program 15 with the following settings: pulse voltage, 1300 V; pulse width, 20 ms; pulse number, 2. The cells were then dispensed into prewarmed RPMI 1640 medium without antibiotics and plated into 24-well plates. Cells were then incubated for 2 days followed by stimulation. Nontargeting and TRAM-, Mal-, and TRIF-targeting siRNAs were purchased as SMARTpool ON-TARGETplus oligonucleotides from Thermo Fisher. Negative and custom siRNAs were synthesized by Qiagen, and the target sequences were as follows: MyD88, 5′-AACTGGAACAGACAAACTATC-3′; TRAF3, 5′-GGAAGATTCGCGACTACAATT-3′; TRAF6, 5′-AACCACGAAGAGATAATGGAT-3′. To assess the knockdown of mRNA after siRNA treatment, levels of mRNA were measured by quantitative real-time PCR using gene-specific primers.

### Quantitative real-time PCR

Total mRNA was isolated using High Pure RNA isolation kit (Roche Applied Science) according to the manufacturer's instructions. RT-PCR was performed using the Moloney murine leukemia virus reverse transcriptase (Promega, WI) according to the manufacturer's instructions. Quantitative real-time PCR was done using the FastStart Universal SYBR Green Master (Roche Applied Science) and performed in an Mx3000P qPCR machine (Stratagene) with the following primers: hβ-actin Fwd, 5′-CGCGAGAAGATGACCCAGATC-3′; hβ-actin Rev, 5′-GCCAGAGGCGTACAGGGATA-3′; hMal Fwd, 5′-AGCTGTCATGCGTTATCTGC-3′; hMal Rev, 5′-TCACTCCAATTTCCGGGGTC-3′; hMyD88 Fwd, 5′-ACCCAGCATTGAGGAGGATTG-3′; hMyD88 Rev, 5′-GAAGGCATCGAAACGCTCAG-3′; hTRAF3 Fwd, 5′-CTCACAAGTGCAGCGTCCAG-3′; hTRAF3 Rev, 5′-GCTCCACTCCTTCAGCAGGTT-3′; hTRAF6 Fwd, 5′-CCTTTGGCAAATGTCATCTGTG-3′; hTRAF6 Rev, 5′-CTCTGCATCTTTTCATGGCAAC-3′; hTRIF Fwd, 5′-GGATCCCTGATCTGCTTGGG-3′; hTRIF Rev, 5′-GAATGTCGAAGGCGCTAGGA-3′; hTRAM Fwd, 5′-TTCCTGCCCTCTTTCTCTCTC-3′; hTRAM Rev, 5′-AACATCTCTTCCACGCTCTGA-3′. Relative mRNA expression was calculated using the comparative *C_T_* method, normalizing the gene of interest to the housekeeping gene β-actin and comparing it with an untreated sample transfected with control siRNA as calibrator.

### Assessment of intracellular bacterial survival

Cells (1 × 10^6^ cells/ml) were seeded into 24-well plates and infected with live *S. aureus* at an MOI of 100. At 30 min post-infection, medium was replaced with medium containing gentamicin (200 μg/ml) to kill extracellular bacteria. In experiments involving inhibitors, cells were pretreated with inhibitors or a DMSO vehicle control for 1 h prior to infection. At the times indicated, infected cells were pelleted, the supernatant was removed, and the cells were lysed by the addition of 100 μl of 0.1% (v/v) Triton X-100. Serial dilutions of the cell lysates were prepared in PBS and plated onto tryptic soy agar to determine cfu/ml. Bacterial intracellular survival was expressed as cfu/ml or as the percentage of intracellular cfu at each indicated time compared with intracellular cfu after 15-min gentamicin treatment (*i.e.* 45 min post-infection).

### Assessment for phagocytosis

To assess the rate of *S. aureus* phagocytosis by THP-1 cells, cells were infected with CFSE-labeled *S. aureus* for 30 min followed by a 15-min treatment with medium containing gentamicin (200 μg/ml) to eliminate extracellular bacteria. Cells were then immediately fixed in 2% (v/v) paraformaldehyde and analyzed on BD FACSCanto II by gating on forward scatter and side scatter, and the percentage of CFSE^+^ cells was assessed as an indicator of intracellular bacteria.

### Statistical analysis

Statistical analysis was carried out using GraphPad Prism statistical analysis software. For protein and mRNA measurements, differences between groups were analyzed by the unpaired Student's *t* test. For intracellular survival, significance was assessed by analysis of variance (ANOVA) with the appropriate post-test and by using repeated measures where required.

## Author contributions

J. M., R. M. M., and A. G. B. conceptualization; J. M., M. E. M., M. M. K., R. M. M., and A. G. B. formal analysis; J. M., M. E. M., M. M. K., and A. G. B. investigation; J. M. and M. E. M. methodology; J. M., M. M. K., and A. G. B. writing-original draft; M. E. M., R. M. M., and A. G. B. writing-review and editing; R. M. M. and A. G. B. supervision; R. M. M. and A. G. B. funding acquisition.

## Footnotes

4 The abbreviations used are:

PAMP: pathogen-associated molecular pattern
BMDM: mouse bone marrow derived macrophage
CFSE: carboxyfluorescein succinimidyl ester
IFN: interferon
IFN-I: type I IFN
IKK: inhibitor of IκB kinase
IL-1: interleukin-1
IRAK: IL-1 receptor–associated kinase
IRF: IFN regulatory factor
LTA: lipoteichoic acid
Mal: MyD88 adaptor-like
MOI: multiplicity of infection
PBMC: peripheral blood mononuclear cell
PRR: pattern recognition receptor
TAK1: transforming growth factor β–activated kinase 1
TBK1: TRAF family member-associated NF-κB activator–binding kinase 1
TLR: Toll-like receptor
TNF: tumor necrosis factor
TRAF6: TNF receptor associated factor 6
TRAM: TRIF-related adaptor molecule
TRIF: Toll/IL-1R domain–containing adaptor inducing IFNβ
FCS: fetal calf serum
ANOVA: analysis of variance.
